# Social value of maintaining baby-friendly hospital initiative accreditation in Australia: case study

**DOI:** 10.1186/s12939-020-01365-3

**Published:** 2021-01-07

**Authors:** Andini Pramono, Julie Smith, Jane Desborough, Siobhan Bourke

**Affiliations:** Department of Health Services Research and Policy, Research School of Population Health, College of Health and Medicine, Australian National University, 63 Eggleston Road, Acton, Canberra, Australian Capital Territory 0200 Australia

## Abstract

**Background:**

Breastfeeding has positive impacts on the health, environment, and economic wealth of families and countries. The World Health Organization (WHO) launched the Baby Friendly Hospital Initiative (BFHI) in 1991 as a global program to incentivize maternity services to implement the Ten Steps to Successful Breastfeeding (Ten Steps). These were developed to ensure that maternity services remove barriers for mothers and families to successfully initiate breastfeeding and to continue breastfeeding through referral to community support after hospital discharge. While more than three in four births in Australia take place in public hospitals, in 2020 only 26% of Australian hospitals were BFHI-accredited. So what is the social return to investing in BFHI accreditation in Australia, and does it incentivize BFHI accreditation? This study aimed to examine the social value of maintaining the BFHI accreditation in one public maternity unit in Australia using the Social Return on Investment (SROI) framework. This novel method was developed in 2000 and measures social, environmental and economic outcomes of change using monetary values.

**Method:**

The study was non-experimental and was conducted in the maternity unit of Calvary Public Hospital, Canberra, an Australian BFHI-accredited public hospital with around 1000 births annually. This facility provided an opportunity to illustrate costs for maintaining BFHI accreditation in a relatively affluent urban population. Stakeholders considered within scope of the study were the mother-baby dyad and the maternity facility. We interviewed the hospital’s Director of Maternity Services and the Clinical Midwifery Educator, guided by a structured questionnaire, which examined the cost (financial, time and other resources) and benefits of each of the Ten Steps. Analysis was informed by the Social Return on Investment (SROI) framework, which consists of mapping the stakeholders, identifying and valuing outcomes, establishing impact, calculating the ratio and conducting sensitivity analysis. This information was supplemented with micro costing studies from the literature that measure the benefits of the BFHI.

**Results:**

The social return from the BFHI in this facility was calculated to be AU$ 1,375,050. The total investment required was AU$ 24,433 per year. Therefore, the SROI ratio was approximately AU$ 55:1 (sensitivity analysis: AU$ 16–112), which meant that every AU$1 invested in maintaining BFHI accreditation by this maternal and newborn care facility generated approximately AU$55 of benefit.

**Conclusions:**

Scaled up nationally, the BFHI could provide important benefits to the Australian health system and national economy. In this public hospital, the BFHI produced social value greater than the cost of investment, providing new evidence of its effectiveness and economic gains as a public health intervention. Our findings using a novel tool to calculate the social rate of return, indicate that the BHFI accreditation is an investment in the health and wellbeing of families, communities and the Australian economy, as well as in health equity.

## Background

Breastfeeding is the natural way and biological standard of infant feeding for all mammals. It has benefits to infant and maternal health, and reduces health inequality, as well as reducing harmful impacts to the environment and decreasing health expenditure attributed to preventable illness. Socioeconomic groups with lower education and income levels are less likely to breastfeed when compared to their higher education and income group peers [1]. Children who are not breastfed have, inter alia, higher rates of obesity, malocclusion and asthma, Sudden Infant Death Syndrome, acute otitis media, type 1 and 2 diabetes and lower intelligence quotients [2–6], while a lack of breastfeeding increases maternal risk of ovarian cancer, breast cancer, type 2 diabetes and osteoporosis [3, 7]. The World Health Organization (WHO) recommends breastfeeding exclusively for the first 6 months of infants’ life, with continued breastfeeding to 2 years and beyond [8]. Despite the benefits, the exclusive breastfeeding rate globally is only 41% [9]. High-income countries such as Australia have shorter breastfeeding duration than low- and middle-income countries, even though breastfeeding has been proven to reduce the risk of sudden infant deaths by more than one third in high-income countries and half of all diarrhea episodes and a third of respiratory infections in low and middle-income countries [10].

Factors associated with low breastfeeding initiation and/or duration include maternal and paternal lower education [11, 12], partners’ negative attitudes towards breastfeeding [13], mother/baby separation after birth [12] and lack of health professionals’ knowledge of breastfeeding [14, 15]. The first few hours and days of an infant’s life are critical to establish breastfeeding. Therefore, WHO launched Ten Steps to Successful Breastfeeding in 1989 and Baby Friendly Hospital Initiative (BFHI) in 1991 to focus on providing a high standard of maternity services to enable every infant to attain the best nutrition standards available. In 2018, WHO revised the Ten Steps to facilitate its system level implementation and sustainability [16]. The revisions are subtle, but meaningful for implementation, with the focus shifted from healthcare staff to parents and families, empowering and enabling women and families to make choices regarding infant feeding method based on information free from conflict of interest [17]. BFHI status is awarded to hospitals that implement consistent high quality and ethical maternity care through the Ten Steps to Successful Breastfeeding policy; while remaining independent from formula companies and their affiliates [18], and is re-assessed every 3 years [19]. UNICEF Australia passed governance of BFHI within Australia to Australian College of Midwives [19]. In 2006, the Australian Baby Friendly Hospital Initiative became the Baby Friendly *Health* Initiative in order to more accurately reflect the expansion of the initiative into community health facilities [19].

Implementing the Ten Steps and achieving BFHI accreditation is essential to ensure quality of maternity care received by mothers and families, regardless of their social, economic, race and religious background. The benefits of implementing Ten Steps and achieving BFHI accreditation and its impact on breastfeeding has been demonstrated in research internationally [20–24], and the cost-effectiveness of BFHI in reducing late neonatal infant mortality rate has been established [25]. In 2012, Australian health ministers encouraged all public and private hospitals to implement the ten steps to successful breastfeeding and to work towards or to maintain their BFHI accreditation [26]. Despite evidence that the BFHI improves the wellbeing of mothers and significantly increases the duration of breastfeeding [20], only 10% of births occur in maternity services that are designated as baby-friendly internationally [27], and only 77 out of 266 maternity services (26%) in Australia are baby-friendly accredited as of 2020 [28]. Several Australian studies showed barriers to BFHI implementation and/or accreditation that are similar to those described internationally [29], such as lack of policy support and funding due to the low priority and value of breastfeeding [30, 31]. Breastfeeding-related programs, including BFHI, has obvious social impacts, yet few studies have examined this. One study has described the social value of a breastfeeding counselling and support program in Nairobi [32] and another described the social value of breastfeeding support group in Ireland [33]. SROI is a novel methodology used to measure social impacts by comparing the investment and outcomes, using monetary values and involving stakeholders. The SROI framework is a valuable tool in this context as breastfeeding-related programs mostly have intangible impacts (e.g. health). By using monetary values, breastfeeding benefits could be compared from economic perspective. One Australian study showed a perception that the cost of BFHI accreditation may outweigh the benefit which could hinder the scale up of the BFHI program in Australia [34]. No studies have explored the social return on investment in BFHI accreditation in the Australian context. So what is the social return to investing in BFHI accreditation in Australia, and does it incentivize maternity and newborn care facilities to gain or maintain BFHI accreditation?

### Research aim

This study aimed to examine the social return on investment (SROI) of maintaining the BFHI accreditation in one public maternity unit in Australia and whether it incentivize maternity and newborn care facilities to gain or maintain BFHI accreditation.

## Methods

### Sample and location

The study was conducted in Calvary Public Hospital, Canberra, an Australian BFHI-accredited public hospital in August 2019. We selected this hospital because three in four Australian mothers give birth in public hospital [35] and this particular hospital has been BFH-accredited for 15 years.

### Data collection

Interviews aimed to elucidate the costs of maintaining the BFHI accreditation. Underpinned by the Social Return on Investment (SROI) framework, and in collaboration with the Director of Midwifery, the Clinical Midwife Consultant, and the Clinical Midwifery Educator, AP, SB, JD and JS developed a structured questionnaire based on the 2018 Ten Steps to Successful Breastfeeding (Ten Steps) which was used for the interview. AP and SB interviewed the Director of Midwifery and the Clinical Midwife Consultant on 21st August 2019 and took one and a half hours. The interviewees were approached through JD’s network and selected purposively for their understanding of the cost of BFHI implementation, accreditation and maintenance. The interview was conducted by AP and SB. AP is an International Board-Certified Lactation Consultant (IBCLC) and a PhD candidate at the Australian National University (ANU); SB is a Health Economist with expertise in using the SROI framework.; JS is an experienced economist with focus in breastfeeding, regulations of markets in mother’s milk and gender analysis of Australia’s taxation and fiscal policies; and JD is a registered nurse and midwife with experience in qualitative research design and implementation. All authors were female.

### Data storage

The interview was audio-recorded and then transcribed verbatim. Data was stored on password protected computer at the university and only accessible to the primary researcher.

### Data analysis

Data was analyzed in excel using the SROI framework, which uses monetary values to measure social, environmental and economic outcomes of change. The SROI is a framework for measuring and accounting for the much broader concept of value; it seeks to reduce inequality and environmental degradation, and improve wellbeing by incorporating social, environmental and economic costs and benefits [36]. The benefits of breastfeeding are associated with a wide range of outcomes including health and social benefits. Therefore, the SROI methodology was relevant to help understand the value created by these programs to inform policy making. The information obtained in the interviews was supplemented with evidence-based estimations from recent high-quality studies that measured the health and cost saving benefits of breastfeeding (Table 1). These were identified through searches of online databases such as Pubmed and Proquest via ANU Library. We used “prevalence” or “incidence”, “rate”, “name of the disease” and “Australia” as keywords, for example “incidence rate of respiratory infection in Australia”. And we used “Odds ratio”, “name of the disease”, “Australia” and “breastfeeding” for the odds ratio of each benefit. We selected studies or reports that were conducted or collected in Australia, otherwise we selected the latest and highest quality international studies using an accepted evidence hierarchy [56].

**Table 1:** Evidence-based estimation to measure the benefits of breastfeeding

Benefits of breastfeeding	Prevalence/Incidence rate	Odds ratio
**Babies**		
Reduce risk in diarrhea	100%	26% [37]
Reduce risk in respiratory infection	14% [38]	18% [5]
Reduce risk in acute otitis media	25% [39]	43% [6]
Reduce risk of Necrotizing Enterocolitis (NEC)	3% [40]	38% [41]
Higher IQ	0.008% [33]	0.21% [33]
Reduce risk in obesity	67% [42]	26% [43]
Reduce risk in type 1 diabetes	0.012% [44]	55% [3]
Reduce risk in type 2 diabetes	5% [45]	35% [46]
Reduce risk in Sudden Death Infant Syndrome (SIDS)	3% [47]	40% [4]
**Mothers**		
Reduce risk in breast cancer	13% [48]	4% [49]
Reduce risk in cardiovascular disease	5% [50]	9% [51]
Ovarian Cancer	1% [52]	24% [53]
Hypertension	12% [54]	12% [51]
Formula cost saving	Breastfeeding initiation rate in Calvary hospital, Australia = 97%	Exclusive breastfeeding rate in Australia = 15.4% [55]

SROI analysis involves a 5-step process: establishing scope and involving stakeholders, mapping outcomes, evidencing and valuing outcomes, establishing impact, and calculating the SROI ratio. Each step is explained in detail below.

## Results

### Establishing scope and involving stakeholders

First, we identified the stakeholders for SROI analysis. For our analysis, implementation of the Ten Steps as a framework for the BFHI involved two main stakeholders: the mother and baby dyad. The mother and baby dyad included as they were identified to derive the greatest benefits from the BFHI accreditation and sufficient evidence was available, and it was feasible to measure and include. While the maternity facility stakeholders could not be included as the evidence was insufficient to be measured.

### Mapping outcomes

Second, we mapped the outcomes for each stakeholder. A theory of change was developed from the literature, representing how the BHFI were expected to bring about change. For mothers, the benefits included risk reduction of breast cancer, cardiovascular disease, ovarian cancer, hypertension, and for no cost related to buying formula [7, 53, 57–59]. For babies, the benefits include reduced risk of diarrhea, respiratory infection, acute otitis media, necrotizing enterocolitis, obesity, Sudden Infant Death Syndrome (SIDS), diabetes, and higher IQ [2, 4, 5, 37, 51, 60–65].

### Evidencing and valuing outcomes

Third, we searched the literature to evidence outcomes (Table 2). The cost in achieving BFHI accreditation based on interview findings (see Appendix). From the interview, costs relating to the BFHI application fee, lunch cost for the assessors, human resource relating to the cost of policy revision, BFHI system monitoring and compliance, breastfeeding counseling, staff training, as well as printing and laminating cost, provision of breastfeeding tools (e.g. nipple shield, pill-cups for cup feeding, hospital-grade breast-pump) and formula purchase for special-needs, preterm and low birth weight babies.

**Table 2:** Financial proxy used to allocate a market price

Babies	Financial proxy	Cost
Reduce risk of diarrhea	Cost of gastrointestinal [66]	AUD 20.27
Reduce risk of respiratory infection	Cost of influenza-related disease [67]	AUD 2864
Reduce risk of acute otitis media	Cost of treating otitis media in Australia [68]	AUD 594
Reduce risk of necrotizing enterocolitis	Cost of NEC treatment [69]	AUD 13,863
Higher IQ	Annual earnings (average weekly income in Australia [33] × 52 weeks)^a^	AUD 89,487
Reduce risk of obesity	Cost of obesity in Australia [70]	AUD 2500
Reduce risk of type 1 diabetes	Cost of diabetes in Australia [71]	AUD 3131
Reduce risk of type 2 diabetes		
Reduce risk of Sudden Infant Death Syndrome (SIDS)	Annual earnings (average weekly income in Australia [33] × 52 weeks)^a^	AUD 89,487
**Mothers**		
Reduce risk of breast cancer	Cost of breast cancer treatment per case in Australia [72]	AUD 36,448
Reduce risk of cardiovascular disease	Cost of cardiovascular disease treatment in hospital in Australia [73]	AUD 1700
Not buying formula	Formula supply for 1 year for full formula-fed baby (1.5 tins for a week for the first 6 months and 0.6 tin for a week for the next 6 months) ^a^ We followed WHO guidance [74] and adapt it to Australian settings	AUD 1160
Reduce risk of ovarian cancer	Cost of ovarian cancer treatment per person in Australia [72]	AUD 31,958
Reduce risk of hypertension	Cost of hypertension treatment per diagnosed case [73]	AUD 570

^a^assumption

### Establishing impact

Deadweight, attribution, and displacement were subtracted from the outcome to reduce the risk of over-claiming benefits [36]. To determine the specific value, we reviewed the literature on breastfeeding. Deadweight relates to a change that would have happened anyway even if BFHI was not implemented [36]; we assumed that 5% of benefits would have happened without the BFHI. Displacement is an assessment of how much of the outcome displaced other outcomes [36]; we assumed the BFHI would displace 20% of other activity. Attribution is the term used for change that occurred caused by other intervention [36]; we assumed 25% of benefits were attributed to other activities. We also assumed that 20% of the benefits would decline (drop off) over time.

### Calculating the SROI and sensitivity analysis

In this step we estimated how long the outcomes will last and used them in the analysis. Here we knew the duration of the outcome due to earlier literature search and interviews. We assumed the benefit included the risk reduction of diarrhea, respiratory infection, acute otitis media and necrotizing enterocolitis lasted for three years; higher IQ, risk reduction of obesity, type 1 and type 2 diabetes and SIDS for 30 years; risk reduction of breast and ovarian cancer, hypertension and cardiovascular disease for 15 years; and formula supply for two years. The costs and benefits were discounted to calculate the net present value, to ensure that the costs and benefits in different time periods were comparable. The recommended rate of 4% [75] was used, recognizing the value of cash today is higher than value of cash in the future. This is the net present value (NPV). After the net present value was calculated, we subtracted the investment and then divided it by the total input, that being the total monetary investment in the BHFI.

$$ \mathrm{SROI}=\frac{Net\  present\ value\ of\ BHFI\ (NPV)- Value\ of\ investment\kern0.5em }{Value\ of\ investment\ } $$

We conducted a sensitivity analysis identifying the estimated with the greatest impact on the SROI ratio, to test how sensitive the ratio is to changes in these estimates including in the deadweight, displacement and attrition and specific estimates.

The average number of births in Calvary public hospital was 1000 annually, with exclusive breastfeeding rate on discharge of 97% in 2019. The value of benefits and costs is summarized in Table 3.

**Table 3:** Value of benefits and costs of BFHI accreditation at Calvary Public Hospital

Benefits	Annual amount in AUD
**Babies**	
Reduce risk of diarrhea	3004
Reduce risk of respiratory infection	41,138
Reduce risk of acute otitis media	36,397
Reduce risk of necrotizing enterocolitis	100,591
Higher IQ	9
Reduce risk of obesity	276,832
Reduce risk of type 1 diabetes	118
Reduce risk of type 2 diabetes	33,106
Reduce risk of Sudden Infant Death Syndrome (SIDS)	612,091
**Mothers**	
Reduce risk of breast cancer	111,668
Reduce risk of cardiovascular disease	4186
Not buying formula	121,859
Reduce risk of ovarian cancer	52,462
Reduce risk of hypertension	4679
**Total value of benefits**	**1,375,050**
**Investments**	
**Total value of investments**	**24,433.80**
**Net Yield (benefits less investments)**	**1,373,705.73**
**Social Return on Investment (SROI)**	**55.38**

The social return (benefits) was calculated to be AU$ 1,375,050 and total investment required was AU$ 24,433 per year. Therefore, the SROI ratio was 55:1, which meant that every AU$ 1 invested in the BFHI generated approximately AU$55 of benefit to the Australian economy. The payback period was 0.63 month, which meant that all the investment would return in around 1 month.

For our baseline estimation of the SROI we used conservative assumptions. We conducted sensitivity analysis by trying different assumptions (Table 4). The SROI calculation was dominated by the high value of risk reduction in obesity and SIDS for babies as well as breast cancer risk reduction for mothers.

**Table 4:** Base and new case scenarios

Sensitivity analysis	Base case	New case	New ratio
Attribution	25%	50%	AU$ 36
Deadweight	5%	50%	AU$ 28
Displacement	20%	0%	AU$ 69
Drop off	20%	50%	AU$ 16
Discount rate	4%	6%	AU$ 51
Value of obesity risk reduction	26%	22%	AU$ 53
		30%	AU$ 57
Value of SIDS risk reduction	40%	18%	AU$ 40
		56%	AU$ 66
Value of breast cancer risk reduction	4.3%	2.9%	AU$ 53
		5.8%	AU$ 57
Total value of outcome	On average AU$ 98,218	Value divided by 2	AU$ 27
		Value multiplied by 2	AU$ 111
Birth type	Single birth (N mother = 1000)	Twins and triplet (N mother = 700)	AU$ 52

All scenarios tested demonstrated the SROI ratio in favor of the BHFI was > 1, indicating that social value of the BHFI is likely to be greater than the investment made in the program.

## Discussion

Our results demonstrate that every investment of AU$1 drives a social return of AU$55. This evaluation also demonstrated the impact of the BHFI whose principal goals are to ensuring maternity service quality is equitable for every mother and family. Other studies have also found a positive social return for breastfeeding support programs; for example, a nutritional counselling and breastfeeding support program in Nairobi brought US$71 for every US$1 invested or AU$71.07 for every AU$1 (US41 = AU$1.35) [32]. A breastfeeding group facilitated by Public Health Nurse in Ireland brought €15.85 for every €1 invested or AU$ 15.81 for every AU$ 1 (€1 = AU$0.061) [33]. The Nairobi study demonstrated a higher SROI than the maintenance of the BFHI accreditation in our study. This difference might be attributed to the fact that the Nairobi study calculated benefits not only for mothers and babies, but also for siblings, fathers, grandmothers, health care providers, community health volunteers, data collectors and day-care centers. The program was facilitated in community setting, which can be less costly to establish and maintain than in hospital settings due to the smaller number of staff and facility overheads. While the Irish study demonstrated lower SROI than our study, this might be attributed to the fact that like our study, it calculated benefits to mothers and babies, but the cost of investment would be less in a community setting.

One strength of the SROI methodology includes deep engagement with stakeholders, enabling practice-based identification of outcomes and values. Difficulties can be encountered in valuing outcomes and what might have happened anyway. This type of research is most commonly conducted by consultants, which can be costly [76]. There are few peer-reviewed reports of SROI in the public domain, limiting our capacity to compare our findings with those from previous studies [76].

Modelling conducted for the Lancet Breastfeeding Series estimates that global economic losses related to lower cognition from not breastfeeding reached a staggering US$302 billion in 2012, equivalent to 0.49% of world gross national income. In high-income countries alone these losses amounted to US$231.4 billion, equivalent to 0.53% of gross national income [10]. The annual cost of not breastfeeding according to WHO recommendations (6 months of exclusive breastfeeding and continued breastfeeding until 2 years old or beyond) globally was approximately US$1.1 billion annually from preventable maternal and infant morbidity and mortality [77]. In the Australian Capital Territory alone, the hospital cost of treating five common but preventable diseases by breastfeeding (gastrointestinal illness, respiratory illness, otitis media, eczema and necrotizing enterocolitis) was estimated at AU$1–2 million annually in 2001 [78]. An American study of suboptimal breastfeeding cost of necrotizing enterocolitis morbidity and mortality in extremely low birth weight newborn calculated US$27.1 million in direct medical costs, US$563,655 in indirect nonmedical costs and US$1.5 billion in cost attributable to premature death [79]. The promotion of breastfeeding is protective of both the health and wealth of society.

As part of our SROI analysis, stakeholder engagement did not provide all the inputs to the SROI model resulting in some of the outcome values being taken from the literature. Compared to conventional Return on Investment analysis, SROI not only calculates benefits against capital invested, but also takes into account externalities (spillover effects from the intervention) [80]. In fact, in the real world there are no activities entirely limited to its direct impacts, as there are consequences which also affect broader social, economic and environmental dimensions [80].

Breastfeeding can play an important role in narrowing health inequalities. Low breastfeeding rates are related to several factors, and exacerbated by disparities including access to services and socioeconomic and educational background of the mother [81, 82]. Pregnancy presents a unique opportunity for a universal population health intervention to reduce social inequalities. As shown by this research embedding breastfeeding support programs, such as the BFHI, into routine care benefits society and contributes significantly to reducing infant and mother health disparities. In a publicly funded health system, like Australia’s, it provides an opportunity to intervene before systemic barriers that create differential experiences for mothers occur [83]. There is overwhelming evidence that the benefits of breastfeeding in both the short and long term enable infants to have the best possible health regardless of family’s social and economic background. Empowering mothers and families with knowledge that breastfeeding provides the ideal nutrition for children could also meet other policy aims of government. A key aim of the Australian government’s closing the gap policy is targeted at improving Aboriginal and Torres Strait Islander health and to halve the gap in child (ages 0–4) mortality rates. Within the indigenous community infant and child mortality is twice as likely before the age of 5, than their non-indigenous counterparts [84]. Research has shown that Indigenous women are less likely to breastfeed their babies [85]. One of the reasons attributed to this decrease is lack of professional support services [86] such as those offered by the BFHI Ten Steps criteria for quality maternal and newborn care.

The perceived lack of policy commitment to BFHI in Australia might be due to low valuation of breastfeeding as a result of the invisibility of breastfeeding and breast milk’s contribution from an economic perspective [82]. Breastfeeding and breast milk are perceived as free products, even though it is not free when it costs mother’s time and effort. One Australian study measured the economic value of the production of human milk (e.g. gross domestic product/GDP) and showed that human milk production levels exceed $3 billion annually [82].

How health care is financed and who benefits from the BHFI impacts the support for BHFI implementation and/or accreditation. Hospital management may not perceive the returns from investing in BFHI accreditation to be high enough if the hospital funding is activity based i.e. on how many cases of illness and disease are treated. Health issues avoided by breastfeeding not only benefits baby and mother, as we measured in this study, but also benefits to the health system and society by avoiding child and mother morbidity and mortality [77, 87]. It also benefits fathers, such as through pride and confidence when his partner and babies get healthiest outcomes [88], which we did not use in our study due to limitations of benefit estimation in the literature. BHFI program success was, in effect, measured through hospital admission avoided, In the Australian health financing context based substantially on case-mix funding, the hospital misses out on payments it may have received had those babies been readmitted for treatment in hospital, such as for gastrointestinal or respiratory infections. This institutional disincentive potentially disadvantages the BHFI’s implementation. A change in funding arrangements, to where a hospital could receive funding for supporting prevention programs such as the BHFI, could offset in full or in part the cost of the program, and possibly encourage further adoption of the BHFI in Australian hospitals.

Supporting mothers in the early days after birth in hospital through the BFHI is essential for health equity, as exclusive breastfeeding in hospital is associated with longer duration of breastfeeding [21, 22, 24, 89], particularly in mothers from lower socioeconomic backgrounds [90]. The BHFI represents an initiative that is available for all, regardless of their socioeconomic status, and it address inequalities throughout the lifetime.

### Implications

The results of our study align with previous research regarding the SROI of breastfeeding programs. Investment in breastfeeding support programs, including the BFHI, benefits the community. As the social benefits are greater than the investment and it provides the best start for every infant, the BFHI needs to be prioritized by the government. In principal, the Australian government supports and promotes breastfeeding, and specifically the BFHI, at the national [86, 91, 92] and state and territory levels [93–95]; however there is lack of follow-up action. The Best Start report recommended the BFHI to be integrated with national accreditation standards since 2007 [86]. The 2010 Australian National Breastfeeding Strategy [96], and its 2019 update included the integration of BFHI into national standards, however neither recommendation has yet been actioned [97]. Our evidence quantifies the value of the BFHI program, identifying economic value for investment. This evidence provides a strong incentive for governments to invest more in motivating, implementing and maintaining BFHI accreditation in all Australian hospitals.

### Limitations

This study measured the benefits compared to the cost invested by the hospital. Nevertheless, our study did not include cost savings for healthcare providers, which were not taken into account due to large gaps in the literature relevant to the Australian health system. There is also no literature on the benefits to healthcare professionals in implementing and/or achieving the BFHI accreditation. Moreover, mothers’ time and effort to breastfeed were not included in the calculation, with only limited data on this important investment available in the current literature [98–100]. The impact of breastfeeding support programs on mothers is well documented [101–103]; however, elucidation of the SROI from mothers’ perspectives would be of great value in further clarifying the social impact of implementing the BFHI. Our examination of the SROI of implementation of the BFHI in one public hospital in Canberra, Australia provides the foundation for future research in other hospitals and community settings.

## Conclusion

The results of our study indicate that implementation of the Ten Steps and the BFHI is worth the investment; the social return received was far greater than the investment. BFHI accreditation is a way to ensure equitable quality maternity care, but lacks incentives for individual facilities in Australia’s health financing system. This study was the first that measured the social return of BFHI accreditation and provides strong evidence to prioritize measures driving wider implementation of the BFHI at a national level.

## Data Availability

The data that support the findings of this study are available from the corresponding author upon reasonable request.

## Appendix

**Table 5:** Cost associated to BFHI accreditation (in Australian Dollar)

Ten Steps	Category	Amount			Per year	Per 3 year
	BFHI Application fee	$ 9380.00	every 3 year		$ 3126.67	$ 9380.00
	Lunch for the assessor	$ 150.00			$ 50.00	$ 150.00
Step 1b	Policy revision	$ 52.00	per hour		$ 26.00	$ 78.00
	Policy communication to parents (printing and laminating cost)	$ 9.19	per pc	Commercial price based on Officeworks	$ 30.63	$ 91.90
Step 1c	BFHI Monitoring system	$ 31.00	per hour	1–2 h/every 6 months [104]	$ 124.00	$ 372.00
Step 2	Staff training group 1	$ 66.00	per person	100 staffs	$ 2200.00	$ 6600.00
	Printing cost for supervised clinical practice record book	$ 2.88	per booklet	Commercial price based on Officeworks	$ 96.00	$ 288.00
Step 3	Educator fee for antenatal class	$ 31.00	per hour	level 2 staff; 2 h duration each class	$ 744.00	$ 2232.00
	Printing cost for educational material (fact sheets)	$ 0.69	per side	Commercial price based on Officeworks	$ 1380.00	$ 4140.00
Step 5	Nipple shield	$ 28.00	each	Commercial price based on Chemist Warehouse	$ 1400.00	$ 4200.00
	Pill-cups for cupfeeding	$ 1.00	each	Commercial price based on Medela	$ 50.00	$ 150.00
	Hospital grade breastpump	$ 244.59	each	Commercial price based on Nursing Angel	$ 81.53	$ 244.59
Step 6	Sucrose 24%	$ 55.00	each	Data from interview	$ 13,750.00	$ 41,250.00
	Formula NAN Pro 1 Gold	$ 16.00	per tin	Data from interview	$ 240.00	$ 720.00
	Formula Pre NAN LBW RTF	$ 48.00	per tin	Data from interview	$ 720.00	$ 2160.00
	1 ml and 2 ml syringe	$ 1.00	each	Data from interview	$ 150.00	$ 450.00
	medicine cups	$ 1.00	each	Data from interview	$ 75.00	$ 225.00
Step 7	Rotary cottage (parents’ accommodation)	$ 40.00	per night	paid by the patients		
	Rooming-in room	$ 362.00 (public patient)	per night	paid by the patients		
		$ 660.00 (private patient)	per night	paid by the patients		
	Recliner for accompanying father in special care nursery	$ 478.00	each	Commercial price based on Amart furniture	$ 159.33	$ 478.00
Step 8	Early feeding cues poster	$ 9.19	per pc	Commercial price based on Officeworks	$ 30.63	$ 91.90
Step 9	Counsel on the risk and use of pacifier and teat bottle			integrated with Step 3		
Step 10	Home visit	$ 31.00	per hour	Level 1 or 2	$ 62,000.00	$ 186,000.00
				Grand Total	**$ 86,433.80**	**$ 259,301.39**
